# Effects of Enhanced Recovery After Surgery in Total Knee Arthroplasty for Patients Older Than 65 Years

**DOI:** 10.1111/os.12441

**Published:** 2019-04-04

**Authors:** Hong‐hui Jiang, Xiao‐fei Jian, Yang‐fan Shangguan, Jun Qing, Liao‐bin Chen

**Affiliations:** ^1^ Department of Orthopedic Surgery Zhongnan Hospital of Wuhan University Wuhan China; ^2^ Department of Orthopedics, the Central Hospital of Wuhan, Tongji Medical College Huazhong University of Science and Technology Wuhan China

**Keywords:** Elderly patient, Enhanced recovery after surgery, Total knee arthroplasty

## Abstract

**Objectives:**

To explore the safety and efficacy of the enhanced recovery after surgery (ERAS) program for elderly total knee arthroplasty (TKA) patients.

**Methods:**

A prospective controlled study was conducted for patients older than 65 years, who would undergo unilateral TKA with a minimum follow‐up of 2 years. Patients were divided into an ERAS group (*n* = 106) and a traditional group (*n* = 141) based on the patients’ willingness to participate in the ERAS program. Baseline parameters of American Society of Anesthesiologists classification and comorbidity were recorded. Complication, mortality, knee function assessment using knee society score and knee range of motion, and perioperative clinical outcomes were compared between the two groups.

**Results:**

There were no significant differences between the two groups in terms of baseline parameters. Although no significant differences were found in postoperative nausea and vomiting, urinary tract infection, deep venous thrombosis, pulmonary embolism, wound delayed healing, superficial infection, and deep infection, there were significantly fewer total complications in the ERAS group (26/106 *vs* 52/141; *P* = 0.039). No significant difference was found in short‐term mortality (1/106 *vs* 3/141; *P* = 0.836) between the two groups. There were no significant differences in preoperative visual analogue scale (VAS), knee society score (KSS), and range of motion (ROM) between the two groups. Lower VAS scores were found in the ERAS group at time of postoperative day (POD) 1 (*P* = 0.012) and POD 5 (*P* = 0.020); no significant differences were observed at time of postoperative month (POM) 1 and final follow‐up. Higher KSS scores were found in the ERAS group at time of POD 1 (*P* = 0.013), and POD 5 (*P* = 0.011), no significant differences were observed at time of POM 1 and final follow‐up. Increased ROM degree was found in the ERAS group at time of POD 1 (*P* = 0.021); no significant differences were observed at time of POD 5, POM 1 and final follow‐up. Decreased intraoperative blood loss (*P* < 0.001), total blood loss (*P* < 0.001), transfusion rate (*P* = 0.004), and length of stay (*P* < 0.001) were found in the ERAS group; no significant differences were found in operative time and hospitalization costs between the two groups.

**Conclusion:**

The ERAS program is safer and more efficacious in elderly TKA patients compared to the traditional pathway. It could effectively relieve perioperative pain and improve joint function, and reduce blood transfusion, length of stay, and total complications without increasing short‐term mortality.

## Introduction

Total knee arthroplasty (TKA) has become one of the most effective treatments for relieving joint pain and improving joint function in patients with knee arthritis^1^. It has been predicted that the total number of TKA performed in the United States will increase to 3 480 000 cases by 2030^2^. With the developments in medicine, TKA patients have higher expectations for surgical outcomes^3^, ^4^. In addition, the financial burden of health care necessitates that medical institutions shorten hospitalization days and reduce complications^5^. Painless surgery and fast recovery are common pursuits of surgeons and TKA patients^6^, ^7^, ^8^. The core idea of enhanced recovery after surgery (ERAS) is to reduce surgical stress response, alleviate pain during perioperative period, reduce the incidence of complications, accelerate functional recovery, and improve patient satisfaction^9^, ^10^. The focus of ERAS in TKA is to improve surgical techniques and to optimize perioperative management, including reduction of trauma and hemorrhage, optimization of pain and blood management, prevention of infection and deep vein thrombosis, and early mobilization^10^, ^11^, ^12^.

Application of the ERAS program in TKA is beneficial for patients, doctors, and hospitals^6^, ^13^, ^14^, ^15^, ^16^. Auyong *et al.* collected data from 252 primary TKA patients and discovered that the length of stay decreased from 76.6 h to 56.1 h after implementation of the ERAS pathway^6^. Decreasing hospital length of stay may reduce hospitalization costs and increase bed turnover. In addition, a large sample clinical study that included 4500 patients reported a substantial reduction in death rates and reduced transfusion requirements with the fast‐track protocol^14^. Jorgensen *et al.* analyzed in‐hospital thromboembolic events through the Danish National Patient Register, and found that the incidence of early thromboembolic events after fast‐track TKA was low^17^. Due to the increasing pressure of medical costs, and because of its proven effectiveness and safety, the outpatient pathway for TKA has become increasingly popular, with patients generally experiencing same day admission and discharge for TKA^18^.

The still open question is whether the ERAS pathway is feasible in elderly TKA patients. Elderly patients with related comorbidities have been found to have increased lengths of stay in hospital, readmissions, and even mortality after major surgery. In Mathijssen's study, multivariable generalized linear mixed models were used to identify potential factors associated with increased hospital stay; older age and American Society of Anesthesiologists (ASA) III/IV were identified as factors^19^. Using multiple linear regression, Holm *et al.* found that age was the only independent predictor of discharge readiness in TKA^20^. Comorbidities, such as diabetes, hypertension, peripheral vascular disease, and chronic obstructive pulmonary disease, are more common in older patients. In addition, elderly patients are more prone to complications, such as postoperative delirium and thromboembolic events^21^. Therefore, advanced age and comorbidity may limit the success of the ERAS pathway for TKA.

The purpose of this prospective controlled study was to: (i) assess the safety of the ERAS program in patients older than 65 years who initially underwent unilateral TKA surgery, with complications and mortality as the primary concerns; (ii) explore whether the ERAS program could benefit elderly TKA patients in regards to knee function and perioperative clinical outcomes; and (iii) promote the ERAS program in elderly TKA patients.

## Materials and Methods

### *Inclusion and Exclusion Criteria*

**Figure 1:**
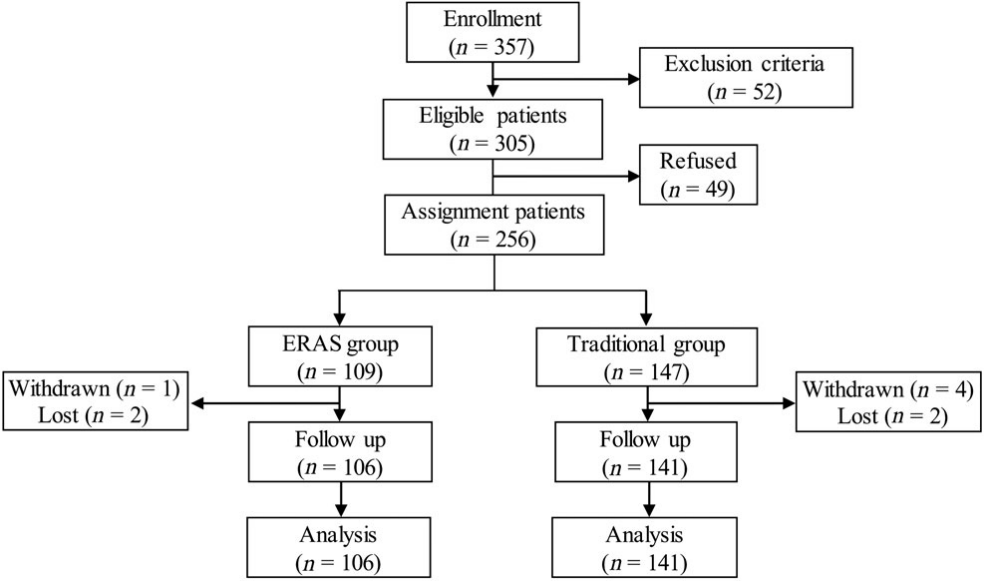
Flow of participants through each stage of the study. ERAS, enhanced recovery after surgery

After approval from the ethics committee of our institute, this prospective controlled study was conducted. Patients were recruited who would undergo unilateral TKA from January 2014 to June 2016 in our hospital. The flow of participants through each stage of the study is presented in Fig. 1.

The inclusion criteria were: (i) elderly patients (>65 years) diagnosed with osteoarthritis or rheumatoid arthritis; (ii) planned primary unilateral TKA; (iii) preoperative hemoglobin ≥10 g/dL and ASA grade I–III; and (iv) hospital discharge with a stabilizing prosthesis implanted. The exclusion criteria were: (i) severe varus or valgus deformity and flexion deformity; (ii) history of knee surgery; (iii) anemia or ASA grade IV–V; and (iv) uncompleted clinical and follow‐up data.

For patients who would undergo TKA surgery, the requirements of the study were explained. Based on the patients’ willingness to participate in the ERAS program or not, patients were divided into ERAS and traditional groups. The pathways of the two groups are presented in Table 1.

**Table 1:** Patient pathway comparison between enhanced recovery after surgery (ERAS) and traditional groups

ERAS pathway	Traditional pathway
**Preoperative interventions**	
Joint function exercise 4 weeks	No joint function exercise
Lung function exercise	No lung function exercise
Oral multimodal analgesia	No oral multimodal analgesia
Preoperative oral carbohydrate loading	No preoperative oral carbohydrate loading
Clear oral fluids up to 2 h before surgery	Clear oral fluids up to 6 h before surgery
**Intraoperative interventions**	
Spinal (preferred) or general anesthetic	General anesthetic
Intravenous dexamethasone	No intravenous dexamethasone
Tranexamic acid	No tranexamic acid
Controlled blood pressure	No controlled blood pressure
Intraoperative avoidance of excessive intravenous fluids	No intraoperative avoidance of excessive intravenous fluids
Active intraoperative warming	No active intraoperative warming
“Cocktail” therapy	No “cocktail” therapy
**Postoperative interventions**	
Multimodal oral analgesia	No multimodal oral analgesia
Early initiation of oral intake	No early initiation of oral intake
Mobilization within 24 h	Mobilization on postoperative day 1

ERAS, enhanced recovery after surgery.

### *Pathway of the Traditional Program*

#### *Preoperative Interventions*

The pathway of the traditional program is presented in Table 1. Before the surgery, the chief surgeon explained the operation method in detail to the patient. To eliminate the patient's fear of pain, pain management measures were also discussed. Preoperative nutrition of the patient was assessed, and hypoalbuminemia was treated with internal medicine. In addition, gastrointestinal motility stimulating drugs were helpful for patients with poor appetite. Clear oral fluids were not allowed at least 6 h before surgery. No preoperative physiotherapy, oral multimodal analgesia, or carbohydrate loading was performed in the traditional group.

#### *Intraoperative Interventions*

Patients in the traditional group underwent general anesthesia. After the tourniquet was used, knee mid‐anterior incisions were made and the medial parapatellar approach was selected. After the knee prosthesis was placed, an indwelling drainage tube was used and the incision was sutured. During the operation, no intravenous dexamethasone, tranexamic acid, or “cocktail” therapy were used. Blood pressure was not controlled and there was no avoidance of excessive intravenous fluids in the traditional group.

#### *Postoperative Interventions*

After returning to the ward, water deprivation was recommended in patients for up to 6 h in the traditional group. Ankle pump exercises (300 times a day) were performed on the first day after the operation, and straight leg raising exercise was started after the drainage tube was removed. The knee joint could bend and be straightened on the third day. Patients were encouraged to walk with the help of auxiliary tools on the fifth day.

To prevent deep venous thrombosis (DVT), rivaroxaban (10 mg a day) was orally administered 6 h after surgery. After the stitches were removed, patients were discharged from hospital. Incision care was strengthened to prevent infections, and the knee joint function exercise and DVT prevention were emphasized through regular telephone interviews.

### *Pathway of the Enhanced Recovery after Surgery Program*

#### *Preoperative Interventions*

The pathway of the ERAS program is presented in Table 1. In addition to preoperative instruction and education, preoperative physiotherapy, preoperative analgesia, and update fasting guidelines were highlighted. The importance of preoperative functional exercise was emphasized to enhance muscle strength and increase joint activity. Patients would start functional training for 4 weeks under the guidance of a rehabilitation physician and nurse during outpatient follow‐up, and exercise programs included quadriceps exercise (15–20 min at a time, 4–5 times a day), knee mobility exercise (20 min at a time, 4–5 times a day), and ankle pump exercise (300–500 times a day). In addition, balloon blowing and coughing exercises were encouraged to improve lung function. Etoricoxib (30 mg a day) was used for pain relief before surgery. Fasting for a long duration keeps the patient in a metabolic stress state, which can cause insulin resistance, and is not conducive to postoperative recovery^22^. Therefore, patients in the ERAS group could have a solid diet up to 6 h before the operation, and oral fluids up to 2 h before the operation if they had no gastrointestinal motility disorder.

#### *Intraoperative Interventions*

In the ERAS group, spinal anesthetic was preferred. Dexamethasone 4 mg and tropisetron 5 mg were administered intravenously before anesthesia induction to prevent postoperative nausea and vomiting (PONV). During the surgical procedure, we minimized the damage to the muscles and ligaments. Blood pressure was controlled to maintain a mean arterial pressure between 60 and 70 mm Hg. Ten minutes before skin incision, 1 g of tranexamic acid was injected intravenously, and 1 g of tranexamic acid was intra‐articularly injected when the incision was closed. Before installing the knee prosthesis, gauze was soaked in a sodium chloride solution of 1:50 000 epinephrine, and then covered on the osteotomy surface to reduce bleeding. After the incision was sutured, a “cocktail” therapy^23^ (0.9% sodium chloride [50 mL] combined with ropivacaine [150 mg], ketorolac [30 mg], and epinephrine [0.1 mg]) was used to relieve postoperative pain around the knee joint; multiple injections using fine needles were performed in the joint capsule and subcutaneously. Intraoperative avoidance of excessive intravenous fluids and active intraoperative warming were performed throughout the operation.

#### *Postoperative Interventions*

After returning to the ward, early initiation of oral intake and early mobilization were encouraged. Patients were permitted to drink water. Patients were instructed to perform ankle pump exercises (300 times a day) immediately after waking from anesthesia. On the first day after surgery, isometric contraction of the quadriceps and straight leg raising exercises (10–20 min at a time, 3–5 times a day) were performed. On the second day, the knee joint began to be bent and straightened. On the third day, patients would walk 100 m with the help of auxiliary tools and entered the toilet independently. The functional exercises were performed regularly for 3 months after surgery. In addition, a postoperative multimodal analgesia protocol, including parecoxib (40 mg a day) and intravenous patient‐controlled analgesia, was adopted in the ERAS group. The measures to prevent DVT after the operation were the same as those applied in the traditional group.

### *Outcome Measures*

The basic characteristics of the included patients were carefully analyzed, including gender, age, body mass index (BMI), operation side of TKA, American Society of Anesthesiologists (ASA) classification, and medical history.

Complications were carefully recorded, including PONV, urinary tract infection, DVT, pulmonary embolism, delayed wound healing, superficial infection, and deep infection. In addition, short‐term mortality was also documented during the 2‐year follow‐up period, including from malignant disease, cerebrovascular accident, myocardial infarct, and pneumonia.

Visual analogue scale (VAS), knee society score (KSS)^24^, and active knee range of motion (ROM) were recorded before the operation, at postoperative day (POD) 1, POD 3, POD5, postoperative month (POM) 1, and final follow‐up. Perioperative clinical outcomes were also evaluated, including operative time, intraoperative blood loss, total blood loss, transfusion rate, and length of stay.

### *Statistical Analyses*

All data were collected and analyzed using IBM SPSS Statistics v.24.0 (IBM, Armonk, NY, USA). The continuous variables that corresponded to normal distribution were expressed as mean ± standard deviation and analyzed by independent sample *t*‐test. Repeated measures such as VAS, KSS, and ROM, *LSD t*‐test was used for comparison between the two groups at each time point. Dichotomous variables were analyzed by *χ* ^2^‐test or Fisher's exact probability method. *P* < 0.05 was considered statistically significantly.

## Results

### *Demographic Data*

A total of 247 elderly patients, who underwent unilateral TKA and completed a minimum follow‐up of 2 years, were finally included in this study. In the ERAS group, there were 106 patients, including 58 women and 48 men, and the mean age at time of surgery was 74.2 ± 6.3 years. In the traditional group, there were 141 patients, including 83 women and 58 men, and the mean age at time of surgery was 75.4 ± 5.9 years. There were no significant differences in baseline parameters between the two groups, including gender, age, BMI, operation side, ASA classification, and medical history (Table 2).

**Table 2:** Baseline parameters of the two groups

Parameters	ERAS group(*n* = 106)	Traditional group(*n* = 141)	*P*‐value
Gender (female/male)	58/48	83/58	0.514
Age (year)	74.2 ± 6.3	75.4 ± 5.9	0.122
BMI (kg/m^2^)	32.1 ± 5.1	31.4 ± 4.5	0.251
Operated side (L/R)	67/39	79/62	0.256
**ASA classification**			
I	20 (18.9%)	25 (17.7%)	0.819
II	58 (54.7%)	82 (58.2%)	0.589
III	28 (26.4%)	34 (24.1%)	0.680
**Medical history**			
Diabetes	29 (27.4%)	37 (26.2%)	0.844
Current smoker	39 (36.8%)	46 (32.6%)	0.495
Hypertension	58 (54.7%)	85 (60.3%)	0.380
Peripheral vascular disease	12 (11.3%)	16 (11.3%)	0.995
COPD	18 (17.0%)	27 (19.1%)	0.662

Values are mean ± standard devation, number of participants, or as otherwise indicated.

ASA, American Society of Anesthesiologists; BMI, body mass index; COPD, chronic obstructive pulmonary disease; ERAS, enhanced recovery after surgery; L, left; R, right.

### *Complications and Short‐term Mortality*

During the 2 years of follow‐up, there were no significant differences in PONV, urinary tract infection, DVT, pulmonary embolism, delayed wound healing, superficial infection, and deep infection between the two groups. However, total complications were significantly decreased in the ERAS group (26/106 *vs* 52/141; *P* = 0.039) (Table 3).

**Table 3:** Perioperative outcomes of the two groups

Parameters	ERAS group(*n* = 106)	Traditional group(*n* = 141)	*P*‐value
**Clinical outcomes**			
Operative time (min)	72.4 ± 13.5	73.5 ± 15.6	0.562
Intro‐operative blood loss (mL)	123.7 ± 26.8	146.4 ± 30.2	<0.001
Total blood loss (mL)	304.2 ± 51.4	421.4 ± 72.5	<0.001
Transfusion rate	18 (17.0%)	47 (33.3%)	0.004
Length of stay (day)	9.6 ± 1.6	11.3 ± 1.9	<0.001
Hospitalization costs ($)	6723 ± 681	6639 ± 671	0.334
**Complications (%)**			
PONV	18 (17.0%)	30 (21.3%)	0.398
Urinary tract infection	4 (3.8%)	9 (6.4%)	0.363
DVT	1 (0.9%)	3 (2.1%)	0.825
Pulmonary embolism	0 (0.0%)	1 (0.7%)	0.886
Wound delayed healing	1 (0.9%)	3 (2.1%)	0.825
Superficial infection	2 (1.9%)	5 (3.5%)	0.696
Deep infection	0 (0.0%)	1 (0.7%)	0.803
Total	26 (24.5%)	52 (36.9%)	0.039

Values are mean ± standard deviation, number of participants, or percentage

PONV, postoperative nausea and vomiting; DVT, deep venous thrombosis.

In terms of short‐term mortality, 1 patient died from cardiovascular disease in the ERAS group. In the traditional group, 3 patients died, including 1 case of malignant disease, 1 of cerebrovascular accident, and 1 of respiratory failure. There was no significant difference in the short‐term mortality between the two groups (1/106 *vs* 3/141; *P* = 0.836).

### *Visual Analogue Scale Score*

**Figure 2:**
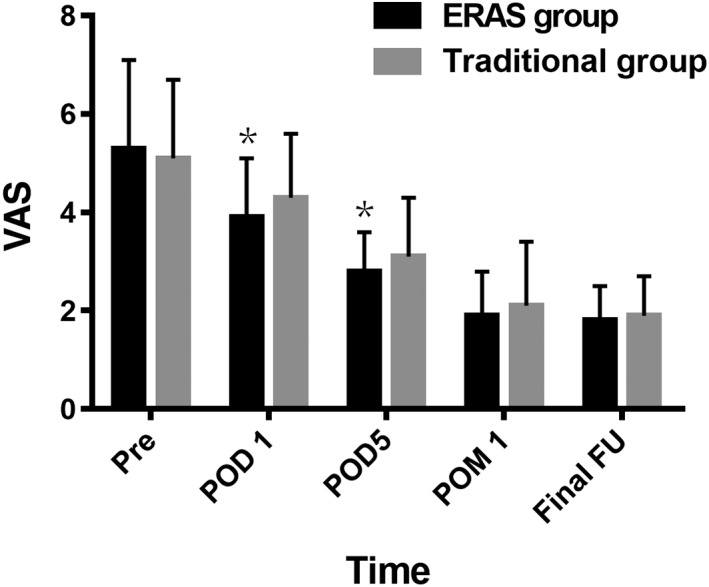
The visual analogue scale (VAS) score of the two groups. ERAS, enhanced recovery after surgery; FU, follow‐up; POD, postoperative day; POM, postoperative month; Pre, preoperation; VAS, visual analogue scale. *P < 0.05.

There was no significant difference in preoperative VAS score between the two groups. Compared to the traditional group, the VAS scores in the ERAS group were significantly lower at time of POD 1 (3.9 ± 1.2 *vs* 4.3 ± 1.3; *P* = 0.012) and POD 5 (2.8 ± 0.8 *vs* 3.1 ± 1.2; *P* = 0.020). There were no significant differences in VAS scores between the two groups at time of POM 1 and final follow‐up (Fig. 2).

### *Knee Society Score*

**Figure 3:**
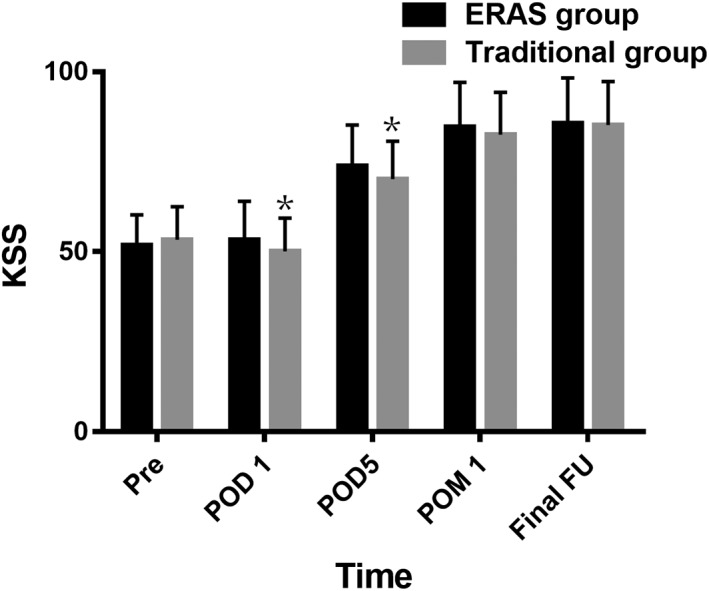
The knee society score (KSS) of the two groups. ERAS, enhanced recovery after surgery; FU, follow‐up; KSS, knee society score; POD, postoperative day; POM, postoperative month; Pre, preoperation. *P < 0.05.

There was no significant difference in preoperative KSS between the two groups. Compared to the traditional group, the KSS in the ERAS group were significantly higher at time of POD 1 (53.3 ± 10.7 *vs* 50.1 ± 9.3; *P* = 0.013) and POD 5 (73.8 ± 11.4 *vs* 70.2 ± 10.5; *P* = 0.011). There were no significant differences in KSS between the two groups at time of POM 1 and final follow‐up (Fig. 3).

### *Range of Motion*

**Figure 4:**
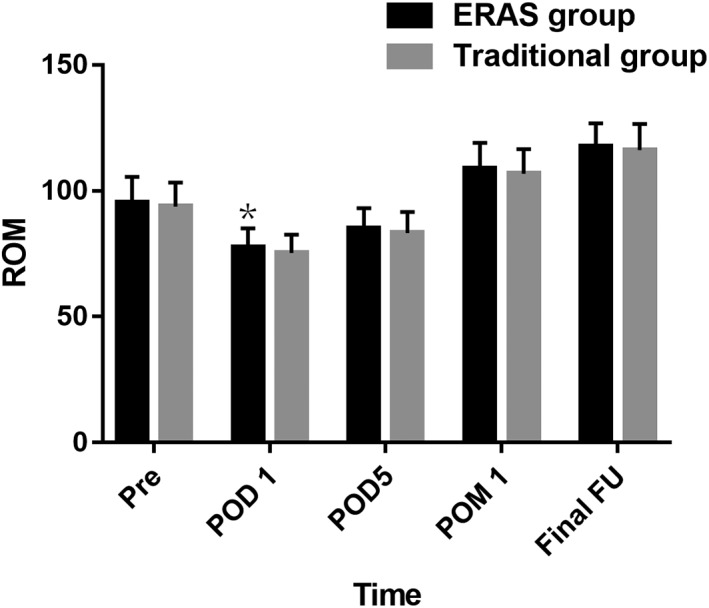
The range of motion (ROM) of the two groups. ERAS, enhanced recovery after surgery; FU, follow‐up; POD, postoperative day; POM, postoperative month; Pre, preoperation; ROM, range of motion. *P < 0.05.

There was no significant difference in preoperative POM between the two groups. Compared to the traditional group, the ROM in the ERAS group was significantly higher at time of POD 1 (77.5° ± 7.6° *vs* 75.3° ± 7.2°; *P* = 0.021). There were no significant differences in ROM between the two groups at time of POD 5, POM 1, and final follow‐up (Fig. 4).

### *Perioperative Clinical Outcomes*

Compared to the traditional group, significantly decreased intraoperative blood loss (123.7 ± 26.8 mL *vs* 146.4 ± 30.2 mL; *P* < 0.001), total blood loss (304.2 ± 51.4 mL *vs* 421.4 ± 72.5 mL; *P* < 0.001), transfusion rate (18/106 *vs* 47/141; *P* = 0.004), and length of stay (9.6 ± 1.6 day *vs* 11.3 ± 1.9 day; *P* < 0.001) were achieved in the ERAS group. There were no significant differences in operative time and hospitalization costs between the two groups (Table 3).

## Discussion

Elderly patients with related comorbidities have increased length of hospital stay, readmissions, and even mortality after major surgery. The still open question is if the ERAS pathway is feasible in elderly TKA patients. This study shows that the ERAS program effectively relieves perioperative pain, and reduces blood loss, length of stay, and complications for patients older than 65 years, who initially underwent unilateral TKA surgery. The study demonstrates the safety and efficacy of the ERAS program in elderly TKA patients.

The ERAS program is safe for elderly TKA patients. Most participants in previous ERAS research studies were middle‐aged, and patients had better basic conditions and lower surgical risk. All the patients included in this study were over 65 years old, which made the ERAS project more challenging. Even though there were no significant differences in PONV, urinary tract infection, DVT, pulmonary embolism, delayed wound healing, superficial infection, and deep infection in the ERAS group, total complications were significantly reduced. In addition, the results indicated that the ERAS program did not increase the risk of short‐term mortality for elderly patients. A large sample study reported that ERAS reduced mortality at 2 years (2.7% *vs* 3.8%), and survival probability up to 3.7 years post‐surgery was significantly better in patients who underwent an ERAS program^25^. The similar short‐term mortality further supports our conclusion that the ERAS program is safe for elderly TKA patients.

The ERAS program can effectively relieve postoperative pain and improve knee function. In this study, early postoperative VAS scores, KSS, and ROM were significantly improved. Multi‐modality analgesia was the core of the ERAS program. A “cocktail” therapy was used in this study, and effectively relieved early postoperative pain^26^, ^27^. Postoperative multimodal analgesia was also beneficial for reducing pain at the surgical site^28^. In addition, the effective pain relief allowed patients to perform early knee functional exercises after surgery, which further improved KSS and ROM. Several studies have reported similar results^8^, ^29^. Zhao *et al.* compared ERAS and traditional treatment in unilateral TKA, and found that postoperative VAS scores, HSS scores, and knee ROM in the ERAS group were significantly lower than in the routine group^11^.

The ERAS program can achieve better clinical outcomes than the traditional protocol. In the ERAS group, the surgeon adhered to the concept of minimally invasive surgery and carefully avoiding unnecessary vessel injury. At the same time, effective hemostasis was achieved after the knee prosthesis was placed. In addition, tranexamic acid was used before skin incision and after incision suturing. Following the above procedural steps, the total blood loss and blood transfusion rate were significantly reduced. Intraoperative blood management is of great significance to patients. Everhart *et al.* found a dose‐dependent relationship between allogeneic red blood‐cell transfusion and surgical site infection risk after arthroplasty, and underlined the need for methods to limit surgical tissue injury, and for optimized blood conservation strategies^30^. Length of stay were also significantly reduced in the ERAS group, which could improve medical efficiency and reduce medical burden^31^.

There are some limitations in this study. First, the ERAS program differed from traditional protocol in several interventions; it was difficult to determine which and how each of the individual changes to the protocol played a role in the observed improvement of patients’ outcome. Second, dexamethasone, tranexamic acid, and multimodal oral analgesia were used in the ERAS group, and adverse drug reactions in elderly patients require further analysis. Third, the study was not a randomized controlled trial, and those who were more understanding of their health condition or who had stronger will to have a fast recovery would choose the ERAS program. This might have caused some selection bias, and further randomized controlled study is needed.

### *Conclusions*

The ERAS program was safe and effective in patients who were over the age of 65 and initially underwent simple unilateral TKA surgery. Implementing the ERAS program relieved perioperative pain and improved joint function, and reduced blood transfusion, length of stay, and total complications without increasing the short‐term mortality.
